# Prognostic significance of the mEPE score in intermediate-risk prostate cancer patients undergoing ultrahypofractionated robotic SBRT

**DOI:** 10.1007/s00066-024-02355-y

**Published:** 2025-01-14

**Authors:** Lucas Mose, Laura Isabel Loebelenz, Alexander Althaus, Maiwand Ahmadsei, Etienne Mathier, Isabelle Broemel, Daniel M. Aebersold, Verena Carola Obmann, Mohamed Shelan

**Affiliations:** 1 Department of Radiation Oncology, Inselspital, Bern University Hospital, University of Bern, 3010 Bern, Switzerland; 2 Department of Diagnostic, Interventional and Pediatric Radiology (DIPR), Inselspital, Bern University Hospital, University of Bern, Bern, Switzerland

**Keywords:** Prostate-specific antigen, Biochemical failure-free survival, Robotic stereotactic body radiotherapy, Extra-prostatic extension, Multiparametric magnetic resonance imaging

## Abstract

**Purpose:**

This study aimed to evaluate the prognostic significance of magnetic resonance imaging (MRI) parameters on biochemical failure-free survival (BFS) in patients diagnosed with intermediate-risk prostate cancer and treated with robotic ultrahypofractionated stereotactic body radiotherapy (SBRT) without androgen deprivation therapy (ADT).

**Methods:**

A retrospective analysis was conducted in patients with intermediate-risk prostate cancer undergoing robotic SBRT delivered in five fractions with a total radiation dose of 35–36.25 Gy. The primary endpoint was biochemical failure as defined by the Phoenix criteria. Among other clinicopathological data, T stage, Prostate Imaging-Reporting and Data System (PI-RADS) score, and multiparametric magnetic resonance imaging-based extra-prostatic extension (mEPE) score were collected and analyzed using the log-rank test.

**Results:**

A total of 74 patients were eligible for analysis. Median age at treatment was 68.8 years and median prostate volume was 47.8 cm^3^. Fifty-four and 14 patients were diagnosed with Gleason scores 7a and 7b, respectively. In total, 40 patients were classified as having unfavorable intermediate-risk prostate cancer according to American Urological Association/American Society for Radiation Oncology/ Society of Urologic Oncology (AUA/ASTRO/SUO) guidelines. The median follow-up was 30 months (range: 4–91.2 months; interquartile range (IQR): 18.5–48 months). The 3‑year BFS was 92%. A total of 12 (16.2%) biochemical failures were reported. In univariate analysis, an mEPE score of 5, the delivered total radiation dose (35 Gy vs. 36.25 Gy), and a prostate-specific antigen (PSA) nadir >1 ng/ml were associated with lower BFS (mEPE–BFS: *p* < 0.001, total radiation dose–BFS: *p* = 0.04, PSA nadir–BFS: *p* =< 0.001).

**Conclusion:**

Patients diagnosed with intermediate-risk prostate cancer with a high mEPE score are more likely to experience biochemical failure after SBRT. Treatment intensification measures, such as administration of concomitant ADT, should be considered.

## Introduction

Prostate cancer is the most common cancer in men and one of the leading causes of cancer-related deaths globally, with around 1.4 million new cases and 375,000 deaths worldwide in 2020 [1]. The majority of prostate cancers are diagnosed in localized stages, making them suitable for potentially curative treatments [2, 3]. Hereby, localized intermediate-risk prostate cancer represents a heterogeneous group, and accurate pretherapeutic differentiation between an organ-confined stage and disease with extra-prostatic extension (EPE) is crucial for treatment decision-making [4]. However, multiparametric magnetic resonance imaging (mpMRI)-based detection of the EPE appears to have limited and heterogeneous sensitivity [4–6]. Therefore, the Prostate Imaging Reporting and Data System (PI-RADS) v2.1 mpMRI features were implemented in a comprehensive MRI-based extraprostatic extension (mEPE) score to predict tumor extension surpassing the prostate capsule [5]. Radiological factors such as abutment of the capsule, tumor–capsule interface length, irregular margins, bulging of the prostatic contour, asymmetry of the neurovascular bundles, and breach of the capsule were implemented in the mEPE score, resulting in a value between 1 and 5 [5]. Individually, these radiological features have low sensitivity (SE) and specificity (SP) for EPE [5]. However, when combined in the mEPE score with a threshold of > 3 or > 4 for a positive diagnosis of EPE, an SE of 0.82 with an SP of 0.77 and an SE of 0.51 with an SP of 0.96 were reported, respectively [5]. For patients undergoing radical prostatectomy, a higher mEPE score was associated with an increased risk of positive postoperative margins and biochemical recurrence [7, 8].

Alternatively, prostate cancer patients may undergo radiotherapy, with options including conventional radiotherapy, moderately hypofractionated radiotherapy, and ultrahypofractionated stereotactic body radiotherapy (SBRT) [9–11]. Given the low α/β ratio of prostate cancer, estimated at around 1.2–1.4, ultrahypofractionated SBRT provides an effective treatment option with a shortened treatment duration of up to 2 weeks compared to conventional or moderately hypofractionated radiotherapy [9–13]. Moreover, American Society of Radiation Oncology (ASTRO), American Society of Clinical Oncology (ASCO), and American Urological Association (AUA) guidelines support the use of ultrahypofractionated SBRT for patients with low- and intermediate-risk prostate cancer [14]. The application of SBRT for high-risk prostate cancer patients shows promising results; however, it remains under debate and cannot be considered the standard of care [15, 16]. In this context, the need for accurate differentiation between organ-confined tumors and those extending over the prostate capsule becomes eminent. Nonetheless, the prognostic significance of mEPE score in patients with intermediate-risk prostate cancer undergoing ultrahypofractionated SBRT remains under investigation. Therefore, this retrospective analysis aims to evaluate the prognostic significance of the mEPE score in intermediate-risk prostate cancer patients treated with ultrahypofractionated robotic SBRT without hormonal treatment.

## Materials and methods

### Study population

**Fig. 1:**
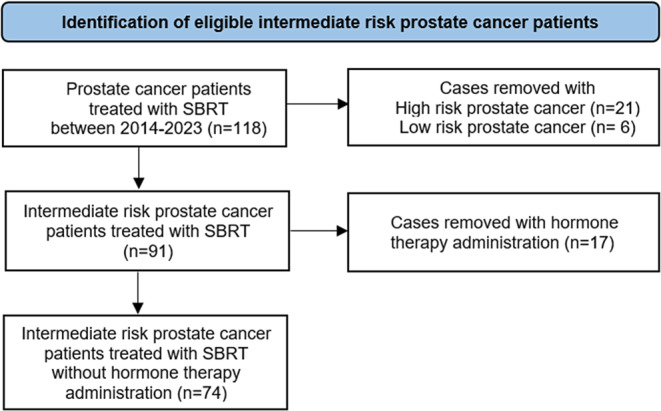
Patient cohort flowchart

After obtaining approval from the regional ethics committee (BE 2022-00120), we retrospectively screened the medical records of 118 patients diagnosed with clinically localized prostate cancer who had undergone robotic ultrahypofractionated SBRT (CyberKnife, Accuracy Inc., Sunnyvale, CA, USA) at Inselspital, Bern University Hospital, Bern, Switzerland, between 2014 and 2023. Inclusion criteria of this single-center retrospective cohort study were intermediate-risk prostate cancer, completion of treatment, and no hormone therapy administration. This resulted in exclusion of 44 and inclusion of 74 patients eligible for analysis. A patient cohort flowchart summarizing this process is displayed in Fig. 1.

All patient- and treatment-related data were retrospectively analyzed in the clinical database. Stratification of intermediate-risk prostate cancer into favorable and unfavorable disease was guided by three risk factors according to the AUA/ASTRO/ Society of Urologic Oncology (SUO) guidelines: prostate-specific antigen (PSA) level 10–< 20 ng/ml, grade group 2 or 3, and clinical stage T2b‑c [17].

Before treatment planning, four intraprostatic fiducial markers were implanted under transrectal ultrasound guidance for intrafractional motion tracking. Two weeks later, patients underwent computed tomography (CT) (1 mm) and contrast-enhanced mpMRI scans with an empty rectum and a full bladder for planning purposes. CT and MRI scans were fused for contouring. Treatment planning was performed using the treatment planning software (Accuray Precision MD Suite version 2.0.1.1). The clinical target volume (CTV) encompassed the prostate and—depending on the risk—the seminal vesicle base (1 cm) was included. A planning target volume (PTV) margin of 3 mm was added posteriorly and of 5 mm in all other directions. The prescribed dose was normalized to the 75–85% isodose. Radiotherapy was delivered every other day in five fractions, with a cumulative dose ranging from 35 to 36.25 Gy. Treatment was delivered using the CyberKnife system.

**Fig. 2:**
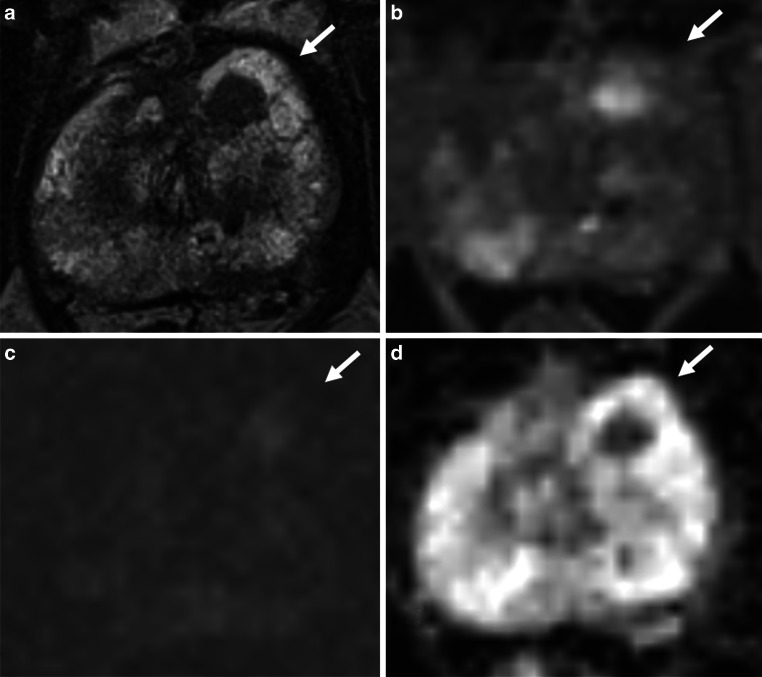
Prostate cancer multiparametric magnetic resonance imaging (mpMRI): mpMRI of a prostate cancer located in the peripheral zone left (indicated by arrow, axial diameter 10 mm), Prostate Imaging Reporting and Data System (PI-RADS) score 4. Absence of all features, resulting in an mpMRI-based extra-prostatic extension (mEPE) score of 1. (mpMRI sequences: **a** T2-weighted, **b** dynamic contrast-enhanced MR perfusion, **c** diffusion-weighted magnetic resonance imaging, **d** apparent diffusion coefficient)

**Fig. 3:**
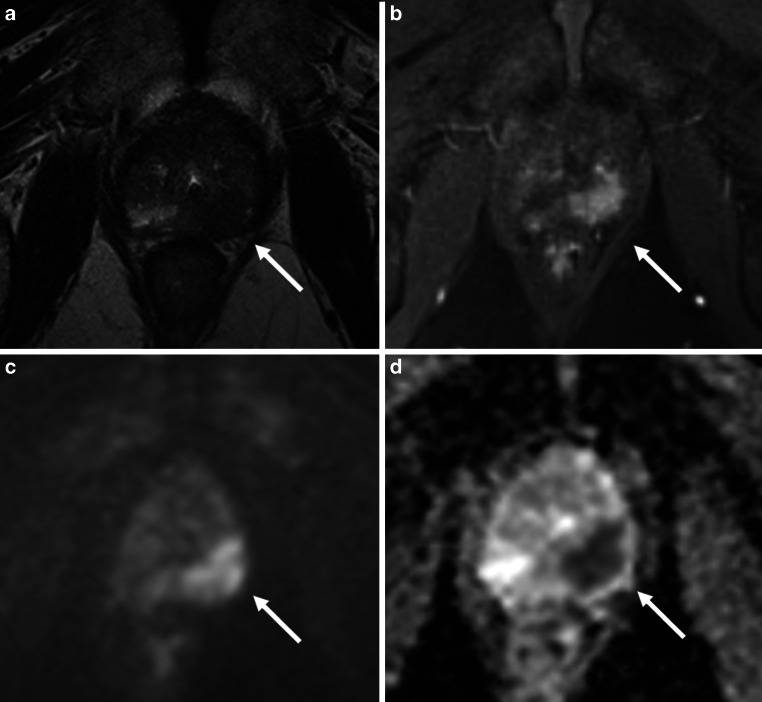
Prostate cancer multiparametric magnetic resonance imaging (mpMRI): mpMRI of a prostate cancer located in the peripheral zone left (indicated by arrow, axial diameter 19 mm), Prostate-Imaging - Reporting and Data System (PI-RADS) score 5. Capsule contact 30 mm, capsule bulging, and irregular capsule margins, resulting in an mpMRI-based extra-prostatic extension (mEPE) score of 5. (mpMRI sequences: **a** T2-weighted, **b** dynamic contrast-enhanced MR perfusion, **c** diffusion-weighted magnetic resonance imaging, **d** apparent diffusion coefficient)

Radiological staging included T stage, PI-RADS, and mEPE score. Lesions scored as mEPE scores 1 and 5 are displayed in Figs. 2 and 3, respectively. In addition, the prostate volume, the presence of dynamic contrast enhancement (DCE) sequences, the number of tumorous lesions within the prostate (single or multiple), and the maximum diameter of the malignant lesion were evaluated. In the case of the two intraprostatic lesions reported in one patient, the lesion with the higher score was considered for statistical analysis. Radiological assessment was conducted by one experienced radiation oncologist and two radiologists, with final scores determined through mutual agreement. According to the European Society of Urogenital Radiology/EAU Section of Urological Imaging (ESUR/ESUI) consensus criteria on mpMRI for the detection of clinically significant prostate cancer, both radiologists are considered experts [18].

### Statistical analysis

The time of biochemical failure-free survival (BFS) was calculated according to the Kaplan–Meier estimate. Differences in BFS were calculated by univariate analysis using IBM SPSS Statistics version: 28.0.1.1 (IBM Corp., Armonk, NY, USA).

## Results

Median age at treatment was 68.8 years (range: 52.2–85.1 years, IQR: 64.1–75.1 years) and median prostate volume was 47.8 cubic centimeters (ccm) (range: 23–93.3 ccm, IQR: 35.6–59.0 ccm). Sixty-eight patients were diagnosed with lesions of Gleason score 7. T‑stages were evaluated, with 22 prostate cancers at cT1c, 24 at cT2a, 11 at cT2b, and 17 at cT2c. Median pre-therapeutic PSA level was 8.5 ng/ml (range: 1.5–18.7 ng/ml; IQR: 5.4–10.8 ng/ml). In total, 34 patients were diagnosed with favorable intermediate-risk and 40 patients with unfavorable intermediate-risk prostate cancer. Clinical, pathological, and treatment characteristics are summarized in Table 1.

**Table 1:** Clinicopathological and treatment characteristics

Patients (*n* = 74)	Median (range)	Interquartile range
**Patient data**		
*Age (years)*	68.8 (52.2–85.1)	64.1–75.1
*Pretherapeutic PSA (ng/ml)*	8.5 (1.5–18.7)	5.4–10.8
*Prostate volume (ccm)*	47.8 (23–93.3)	35.6–59.0
*T stage (n)*		
cT1c	22 (29.7%)	–
cT2a	24 (32.4%)	–
cT2b	11 (14.9%)	–
cT2c	17 (23%)	–
*Gleason score (n)*		
6	6 (8.1%)	–
7a	54 (73%)	–
7b	14 (18.9%)	–
*Risk group (n)*		
Favorable intermediate^a^	34 (45.9%)	–
Unfavorable intermediate^a^	40 (54.1%)	–
**Treatment data**		
*Isodose line (%)*	79.8% (78.2%–80%)	80%–80%
*Radiation dose (n)*		
35 Gy	49 (66.2%)	–
36.25 Gy	25 (33.8%)	–

^a^According to American Urological Association (AUA)/American Society of Radiation Oncology (ASTRO)/Society of Urologic Oncology (SUO) Guidelines [9]

Fifty-one patients had one and 23 patients had two intraprostatic lesions. The median length of the tumor–capsule interface was 14 mm (range: 0–46 mm; IQR: 8–19 mm). In 33 cases, irregular or speculated capsule margins were reported, 48 showed capsule abutment, and 34 bulging of the prostate contour with or without asymmetry of the neurovascular bundles. This led to a total of 10, 29, 22, and 36 prostatic lesions defined as mEPE score 1, 2, 4, and 5, respectively. Sixty-one and 33 lesions were classified as PI-RADS 4 and 5, respectively. Details on radiological assessment are summarized in Table 2.

**Table 2:** Radiological characteristics

*Lesions, n (N* *=* *97)*				
Single intraprostatic lesion	51 (68.9%)			
Two intraprostatic lesions	23 (31.8%)			
*Location, n (zone)*				
Peripheral zone	79 (81.4%)			
Transitional zone	18 (18.6%)			
*Location, n (level)*				
Apex	46 (47.4%)			
Midlevel	43 (44.3%)			
Base	8 (8.2%)			
*DCE positivity, n*				
Yes	95 (97.9%)			
No	2 (2.1%)			
*Prostate volume (cm*^*3*^*), median (range): 47.8 (23.0-93.3)*				
*Lesion diameter (mm), median (range)*	17.6 (6–46)			
*Length of tumor–capsule interface (mm), median (range)*	14 (0–46)			
*Capsule contact, n*				
Abutment	38 (39.2%)			
Spiculated or irregular margins	33 (34%)			
Bulging	34 (35.2%)			
*Radiological scores, n*	mEPE	T2 score	DWI score	PI-RADS
1	10 (10.3%)	0 (0%)	0 (0%)	0 (0%)
2	29 (29.9%)	1 (1%)	0 (0%)	0 (0%)
3	0 (0%)	1 (1%)	1 (1%)	0 (0%)
4	22 (22.7%)	63 (64.9%)	63 (64.9%)	61 (62.9%)
5	36 (37.1%)	31 (32.0%)	31 (32.0%)	33 (34.0%)

*DCE* dynamic contrast enhanced, *DWI* diffusion-weighted imaging, *mEPE* multiparametric magnetic resonance imaging-based extraprostatic extension

**Fig. 4:**
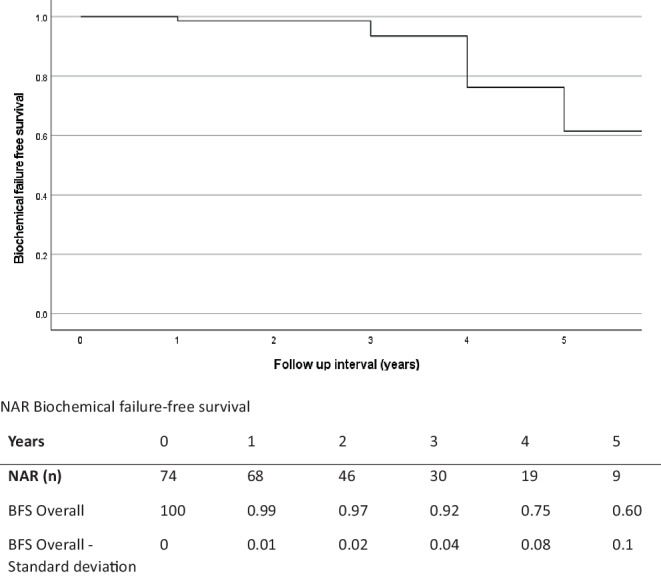
Biochemical failure-free survival (BFS) with numbers at risk (NAR)

Median follow-up was 30 months (range: 4–91.2 months; IQR: 18.5–48 months). Median PSA nadir was 1.0 ng/ml (range: 0.0–5.9 ng/ml; IQR: 0.3–2.5 ng/ml). A benign PSA bounce was seen in 15 patients (20.3%). A total of 12 (16.2%) biochemical failures were reported. Of these, all patients underwent prostate-specific membrane antigen (PSMA) directed positron emission tomography/computed tomography (PET/CT) examinations and a total of 12 recurrences were diagnosed, encompassing five intraprostatic recurrences and seven recurrences involving lymph nodes or distant metastases. The biochemical failure-free survival at 36 months was 92%. Follow-up data are summarized in Table 3 and Fig. 4.

**Table 3:** Follow-up data

*Follow-up*	*Median (range)*	*Interquartile range*
Follow-up (months)	30 (4–91.2)	18.5–48
PSA nadir (ng/ml)	1.0 (0.0–5.9)	0.3–2.5
*PSA*		
Benign PSA bounce, *n/N *(%)	15/74 (20.3)	
Biochemical failures, *n/N *(%)	12/74 (16.2)	
*Location of recurrences, n*		
Intraprostatic recurrence	5	
Lymph node/distant metastases	7	

*PSA* prostate-specific antigen

**Fig. 5:**
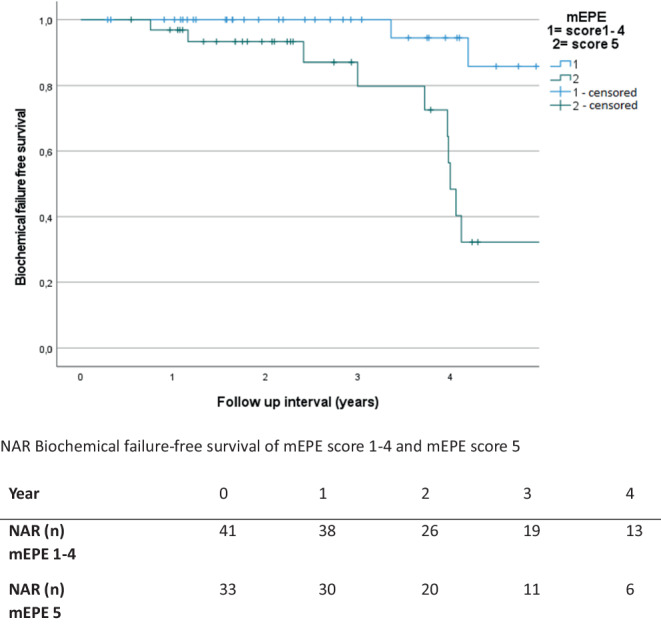
Biochemical failure-free survival of multiparametric magnetic resonance imaging based extra-prostatic extension (mEPE) score 1–4 and mEPE score 5 with numbers at risk (NAR)

**Fig. 6:**
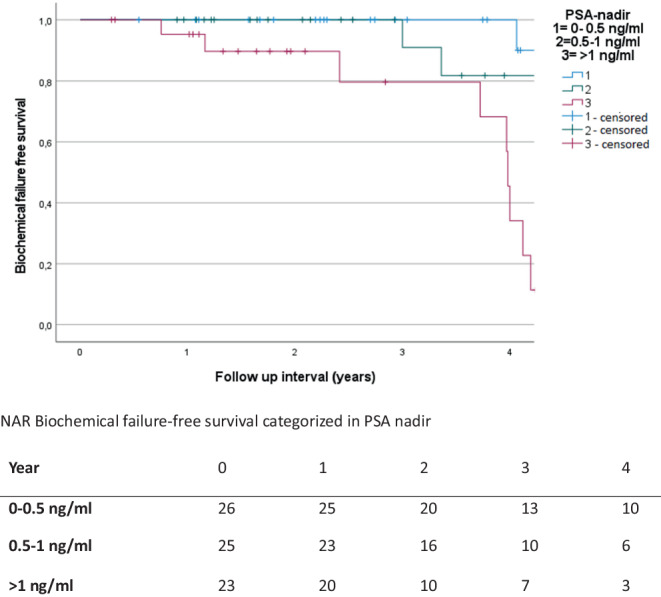
Biochemical failure-free survival with different levels of prostate-specific antigen (PSA) nadir and numbers at risk (NAR)

**Fig. 7:**
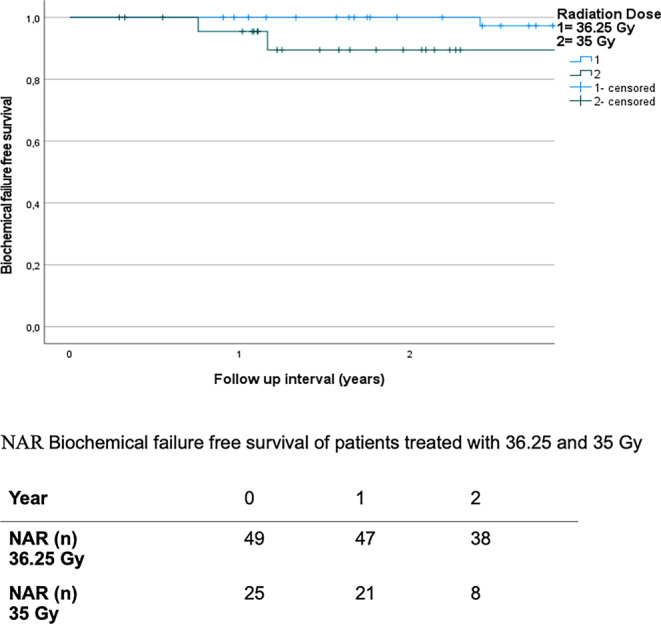
Biochemical failure-free survival of patients treated with 36.25 and 35 Gy with numbers at risk (NAR)

In univariate analysis, higher mEPE score, escalated radiation dose, and higher PSA nadir showed a significant correlation with lower BFS (univariate: mEPE score 1–4 vs. score 5: *p* < 0.001; total radiation dose 35 Gy vs. 36.35 Gy: *p* = 0.04; PSA nadir: reference 0–0.5 ng/ml vs.0.5–1 ng/ml: *p* = 0.698; vs.> 1 ng/ml: *p* < 0.001). Other factors were not significantly correlated with lower BFS. Since the mEPE score was the only significant factor available from pretreatment MRI, no multivariate analysis was performed. Results of the analysis are summarized in Table 4 and Figs. 5, 6 and 7.

**Table 4:** Results of the univariate analysis

Univariate analysis—biochemical failure-free survival (*p*-value)			
*T stage*	*cT2a*	*cT2b*	*cT2c*
Reference: cT1c	*0.704*	*0.736*	*0.609*
*Gleason score*	*Gleason 7a*		*Gleason 7b*
Reference: Gleason 6	*0.628*		*0.487*
*Pretherapeutic PSA*	*>10* *ng/ml*		
Reference: < 10 ng/ml	*0.648*		
*Risk group*	*Unfavorable Intermediate risk*		
Reference: favorable Intermediate risk	*0.719*		
*Prostate volume*	*>42* *ccm*		
Reference: < 42 ccm	*0.791*		
*PI-RADS*	*PIRADS 5*		
Reference: PI-RADS 4	*0.876*		
*Number of intraprostatic lesions*	*Single intraprostatic lesion*		
Reference: two intraprostatic lesions	*0.719*		
*Diameter of intraprostatic lesion*	*>/= 15.5* *mm*		
Reference: < 15.5 mm	*0.533*		
*mEPE score*	*mEPE Score 5*		
Reference: mEPE score 1–4	*<0.001*		
*Radiation dose*	*36.25* *Gy*		
Reference: 35 Gy	*0.04*		
*PSA nadir*	*0.51–1* *ng/ml*		*>1* *ng/ml*
Reference: 0–0.5 ng/ml	*0.698*		*<0.001*

*mEPE* multiparametric magnetic resonance imaging-based extraprostatic extension, *PI-RADS* Prostate Imaging-Reporting and Data System, *PSA* prostate-specific antigen

## Discussion

To the best of our knowledge, this is the first study reporting the prognostic significance of mEPE score in patients diagnosed with intermediate-risk prostate cancer treated with robotic ultrahypofractionated SBRT without ADT. Our findings suggest that a higher mEPE score is associated with lower BFS.

Multiple studies have demonstrated the feasibility and efficacy of ultrahypofractionated SBRT for localized low- and intermediate-risk prostate cancer, showing long-term BFS rates ranging from 85 to 100% [10, 19–21]. Hereby, treatment choices, including decisions on the delivered radiation dose and target volumes, are influenced by whether or not the disease is confined to the organ [2, 22]. Therefore, accurate pretherapeutic imaging is crucial, and mpMRI has been shown to have a significant impact on staging when performed additionally to conventional diagnostics [4, 23]. However, in a meta-analysis by de Rooij et al., MRI-based detection of the extraprostatic extension appears to have limited and heterogeneous sensitivity (SE: 0.61, 95% CI 0.54–0.67) [6]. This may impose a challenge, as SBRT for high-risk prostate cancer remains underinvestigated and is not routinely advised [15, 24]. Moreover, intensified systemic treatment in high-risk prostate cancer patients undergoing radiotherapy is part of the multimodal treatment concept [2].

For patients undergoing prostatectomy, the mEPE score is of relevance, as higher scores are associated with an increased risk of positive postoperative margins and biochemical recurrence [7, 8]. To improve the prediction of side-specific EPE, Soeterik et al. developed a comprehensive nomogram. This model incorporated digital rectal examination staging, the highest ipsilateral International Society of Urological Pathology biopsy grade, and PSA density, in addition to an MRI-based score, achieving a remarkable area under the curve of approximately 0.8 [25]. The additional diagnostic value of combining clinical nomograms with MRI-based models was validated by a systematic review conducted by Zhu et al., which analyzed multiple nomograms for predicting EPE. The combined nomograms may enhance decision-making protocols for prostate cancer patients in the pretreatment phase [26]. Nevertheless, there is no evidence for the impact of mEPE score on the outcome of patients diagnosed with intermediate-risk prostate cancer treated with ultrahypofractionated SBRT.

In this analysis, a higher mEPE score was correlated with lower BFS. A possible explanation is that among patients with higher mEPE scores, some may be understaged and present with EPE, as patients with higher mEPE scores are more likely to exhibit pathological EPE [5]. Consequently, some of these lesions could have belonged to the group of high-risk prostate cancer with T3 stage [17]. This aligns with the findings of Ma et al., who reported understaging of T3 prostate cancer in pretherapeutic MRI compared to pathological findings in 44% of patients undergoing radical prostatectomy [27]. Correspondingly, BFS in our cohort, with 92 and 75% after 3 years and 4 years, respectively, is considerably lower than then BFS stated in the PACE B trial [28]. Hereby, van As et al. reported a 5-year BFS of 95.8% for patients with localized prostate cancer treated with SBRT [28]. This BFS difference could be due to several factors: a total of 7.5% of the patients treated with SBRT in the PACE B trial were diagnosed with low-risk prostate cancer. Furthermore, no patients with Gleason 7b were included [28]. In contrast, low-risk prostate cancer patients were excluded from our analysis, and we included patients with Gleason 7b, accounting for 18.9% of our cohort. These factors have been associated with lower BFS, as stated in an analysis of 477 prostate cancer patients treated with SBRT by Katz et al. [29]. Hereby, BFS of patients with Gleason 7b was comparable to BFS in our cohort [29]. Moreover, 66.2% of our patients were treated with a total radiation dose of 35 Gy, which is lower than the 36.25 Gy total dose applied in the PACE B trial [28]. This may contribute to inferior BFS in our cohort, as dose escalation from 35 to 37.25 Gy in SBRT for low- and intermediate-risk prostate cancer was associated with improved BFS in a prospective trial by Moore et al. Failure rates at 8 years post-treatment were 15% for the 35 Gy dose and 3.4% for the 37.25 Gy dose [10]. Aligning with these findings, a significant BFS difference was observed in our cohort, favoring the total radiation dose of 36.35 Gy over 35 Gy (*p* = 0.04). Additionally, the BFS rates of our cohort patients with high mEPE scores are comparable to the BFS rates of high-risk prostate cancer patients treated with ultrahypofractionated SBRT only published by the SHARP consortium [15]. Therefore, considering the lower BFS and the risk of EPE, a higher mEPE score might be considered an additional risk factor in prostate cancer. Therefore, treatment intensification for this patient subgroup should be considered.

Several approaches for treatment intensification in localized prostate cancer focus on escalating the dose to the visible tumor identified on MRI, as local recurrences typically originate from these areas [30–33]. In the Focal Lesion Ablative Microboost in Prostate Cancer (FLAME) randomized phase III trial by Kerkmeijer et al., patients received up to 77 Gy to the prostate, with an experimental arm receiving an additional simultaneous integrated focal boost to the intraprostatic lesion visible on MRI with up to 95 Gy [31]. Results showed a BFS difference favoring the experimental arm (hazard ratio 0.45, 95% CI 0.28 to 0.71; *p* < 0.001), with no significant difference in acute or late genitourinary and gastrointestinal toxicity [31]. However, the 7% difference in BFS at 5‑year follow-up may also result from the higher rate of patients receiving long-term androgen deprivation therapy (ADT) in the experimental arm (experimental arm 34% vs. standard arm 29%) [34]. Furthermore, Hannan et al. investigated dose escalation in SBRT to the whole prostate with up to 45 to 50 Gy and showed excellent local control with increased severe late toxicities [35]. Accordingly, studies on dose escalation in conventional radiotherapy for intermediate-risk prostate cancer also suggest that dose escalation might have a beneficial impact on BFS and metastases-free survival, with equivalent or slightly elevated toxicities [4]. However, due to limited follow-up, an overall survival benefit through dose intensification remains investigational [4].

In accordance with the above, treatment intensification by adding short-term ADT to SBRT for intermediate-risk cancer patients also yields a BFS benefit but, for the subgroup of patients with unfavorable intermediate-risk prostate cancer, an overall survival (OS) benefit can be obtained [4, 33, 36].

Since none of the patients in this cohort received ADT, and a high mEPE score may indicate a risk of EPE in some cases, treating these patients exclusively with ultrahypofractionated SBRT could lead to undertreatment. It may be reasonable to consider escalating systemic treatment to long-term ADT, since EPE is a high-risk factor and large randomized prospective studies have reported superior OS with long-term ADT over short-term ADT in locally advanced prostate cancer patients [37, 38]. A randomized, prospective trial by Nabid et al. compared OS rates in high-risk prostate cancer patients receiving ADT for 18 months to those receiving ADT for 36 months, with no significant difference in OS found [39]. Therefore, at least 18 months of ADT could be a valid option for such patients.

Another approach could be to switch to a longer fractionation scheme in patients with a high mEPE score, as ultrahypofractionated SBRT for T3 stage prostate cancer is a topic of current debate [15]. Given the increased risk of seminal vesicle invasion in prostate cancer with T3a stage, compared to the T1 or T2 stages, the CTV may be expanded to include portions of the seminal vesicles for patients with a high mEPE score [40, 41]. This approach aligns with the recommendations outlined in the European Society for Radiotherapy and Oncology-Advisory Committee for Radiation Oncology Practice (ESTRO/ACROP) guidelines for the delineation of localized high-risk prostate cancer [40]. However, the proposed treatment intensifications for patients with a high mEPE score but who are classified as intermediate-risk prostate cancer could lead to overtreatment. Therefore, prospective studies are needed to validate these approaches.

According to our institutional policy, we discuss the benefit as well as possible side effects of short-term ADT with each patient. We particularly recommend additional systemic treatment for patients with unfavorable intermediate-risk prostate cancer undergoing ultrahypofractionated radiotherapy. Nevertheless, in cases where patients decline systemic treatment due to potential side effects, such as decreased libido and sexual dysfunction, hot flashes, and fatigue, ADT is omitted, as previously described in similar patient cohorts [28, 29, 42, 43]. However, given the lower BFS rates, we nowadays emphasize the importance of ADT according to established guidelines, especially for unfavorable intermediate-risk prostate cancer patients [2].

In our cohort, the PSA kinetics remain in alignment with the analysis of Jiang et al. [44]. A higher level of the PSA nadir was a significant predictor for biochemical failure in both studies [44]. However, the PSA nadir reported in our cohort is higher than in comparable cohorts [44]. A potential factor contributing to the elevated PSA nadir in our cohort may be the lower radiation dose, as a reduced radiation dose was associated with elevated PSA nadir levels [45]. Moreover, the multi-institutional analysis by Jiang et al. reported a median time to PSA nadir of 40 months. In contrast, our median follow-up was 30 months, suggesting that a significant proportion of patients may not have reached their PSA nadir during the observation period [44]. Moreover, a PSA bounce was noted in 20% of patients, which corresponds with previous literature on PSA bounce after SBRT, reported to range between 17 and 31% [29, 44, 46–48].

In contrast to other studies that reported a divergence between BFS and clinical failure-free survival, we observed a clinical manifestation for every biochemical failure [35, 49, 50]. This difference may be due to our use of PSMA-PET/CT for recurrence diagnosis, which yields superior detection rates for recurrences. Other studies did not report using PSMA-PET/CT [35, 49–52]. Although intraprostatic recurrences predominantly occur in the region of the primary tumor, we observed only two such lesions in that area, which may be attributed to the small number of intraprostatic recurrences [32].

Emerging techniques, such as radiomics models, hold the potential for identifying high-risk lesions within tumors as well as for detecting EPE in prostate cancer patients [53, 54]. In a recent systematic review by Ponsiglione et al., the pooled area under the curve for radiomics-based EPE diagnosis in prostate cancer patients was reported to be 0.8. However, the review highlighted significant heterogeneity among the studies (84.7%; *p* < 0.001) and addressed the lack of independent validation [53]. Moreover, radiomics techniques face multiple challenges, including the standardization of scanner parameters, post-processing algorithms, and applications across institutions. Due to the potential impact on the results, these challenges must be addressed before implementation in clinical practice [53, 54]. Additionally, unlike MRI diagnostics, radiomics models involve poorly explained parameters, while MRI provides the advantage of comparable applicability across institutions [53].

Nevertheless, there are limitations to our results, as the sample size is small, and the analysis is retrospective. Data were collected from one institution only, which may lead to sampling bias, and the follow-up only encompasses a median of 30 months. Additionally, it could be argued that prior biopsy and fiducial marker implantation introduced uncertainties into the MRI reading, as each intervention requires penetration of the capsule tissue. This may influence factors assessed in the mEPE score, such as the irregularity of capsule margins. Therefore, validation of our results in larger cohorts with longer follow-up periods is still needed.

## Conclusion

This study suggests that in patients with intermediate-risk prostate cancer treated with ultrahypofractionated robotic SBRT, a higher mEPE score is associated with lower BFS. Hence, ultrahypofractionated SBRT alone could result in undertreatment, clinicians and patients must be aware that omission of ADT might compromise the oncological outcome in the presence of a higher mEPE score.
